# A Review of Coating Materials Used to Improve the Performance of Optical Fiber Sensors

**DOI:** 10.3390/s20154215

**Published:** 2020-07-29

**Authors:** Changxu Li, Wenlong Yang, Min Wang, Xiaoyang Yu, Jianying Fan, Yanling Xiong, Yuqiang Yang, Linjun Li

**Affiliations:** 1 Department of Physics, School of Science, Harbin University of Science and Technology, Harbin 150080, China; lichangxu@hrbust.edu.cn (C.L.); wangmin@hrbust.edu.cn (M.W.); xyling1964@hrbust.edu.cn (Y.X.); yangyuqiang@hrbust.edu.cn (Y.Y.); 2 School of measurement and communication engineering, Harbin University of Science and Technology, Harbin 150080, China; yuxiaoyang@hrbust.edu.cn (X.Y.); fanjianying@hrbust.edu.cn (J.F.); llj7897@hrbust.edu.cn (L.L.)

**Keywords:** optical fiber sensor, polydimethylsiloxane (PDMS), Mach–Zehnder interference (MZI), photonic crystal fiber (PCF), fiber Bragg grating (FBG)

## Abstract

In order to improve the performance of fiber sensors and fully tap the potential of optical fiber sensors, various optical materials have been selectively coated on optical fiber sensors under the background of the rapid development of various optical materials. On the basis of retaining the original characteristics of the optical fiber sensors, the coated sensors are endowed with new characteristics, such as high sensitivity, strong structure, and specific recognition. Many materials with a large thermal optical coefficient and thermal expansion coefficients are applied to optical fibers, and the temperature sensitivities are improved several times after coating. At the same time, fiber sensors have more intelligent sensing capabilities when coated with specific recognition materials. The same/different kinds of materials combined with the same/different fiber structures can produce different measurements, which is interesting. This paper summarizes and compares the fiber sensors treated by different coating materials.

## 1. Introduction

With the wide application of optical fiber sensors in industrial production and environmental detection, the advantages of optical fiber sensors have been fully reflected. In order to meet the requirements of different applications and improve the performance of optical fiber sensors, a variety of sensors with different structures have emerged. However, the processing of the fiber structure cannot fully develop the potential of the fiber sensor. In the context of the emergence of various optical materials and the gradual perfection of optical fiber sensing theory, various optical materials are known and applied to optical fiber sensors.

For basic fiber sensors, a variety of structures based on MZI (Mach–Zehnder Interference), PCF (photonic crystal fiber), FBG (fiber Bragg grating), and F-P (Fabry–Pérot) are made. Sensors are assigned different tasks depending on the workplace and the accuracy of the test. In temperature sensing, the effective length and refractive index of the optical fiber sensor will change with temperature fluctuation. However, the coefficients of thermal expansion and thermo-optic of SiO_2_ are small, and the overall thermal sensitivity of the fiber sensor is relatively low. To improve the sensitivities of the sensors, many materials with a higher thermo-optical coefficient are coated on the fiber. Sensors coated with polydimethylsiloxane (PDMS), polyimide, UV-sensitive materials, or graphene were proposed to be used with various optical fiber structures. The same fiber sensor may have the ability to simultaneously measure multiple parameters. Similarly, the same optical fiber structure coated with different materials will have different response characteristics. Therefore, it is necessary to analyze the sensing principle of various sensors.

In this review, studies are summarized according to the types of utilized materials and sensing principles. In the first part of this review, we summarize the optical fiber sensors coated with PDMS and discuss them according to the optical fiber structure. At the same time, the advantages of PDMS as a coating material and its sensing function in different applications are introduced. In the second part of the review, the role of polyimide as a coating material in humidity sensing is summarized. In the third part of the review, the different applications of UV-sensitive materials in optical fiber sensing are briefly introduced. In the rest, the performance of graphene, metal ions and other coating materials in sensing is described.

## 2. Sensors Coated with the PDMS

In the development of PDMS, the preparation and performance testing of PDMS films are indispensable steps. Through the discussion of the fabrication method and properties of the PDMS membrane, a kind of film that can be loaded and unloaded repeatedly was prepared [1]. Due to its excellent biocompatibility and biologically relevant mechanical properties, it has the potential to be a fiber coating material. In the fabrication of integrated optical circuits and circuits [2,3,4,5,6,7,8,9,10,11,12,13], PDMS is usually made into a special waveguide in an integrated system [14,15]. After a large number of experimental studies, it was found that the integrated system prepared by PDMS has the characteristics of low loss, high temperature stability, and sufficient mechanical stability, and the sensitive characteristic of temperature and humidity also began to be concerned. At the same time, many optical fiber waveguides directly made of PDMS were prepared [16,17,18]. Due to the good plasticity of PDMS, optical waveguides can easily be fabricated into a variety of different structures, such as knotted, twisted, and tapered fiber. In Table 1, the thermal expansion coefficients of common materials are shown. It can be found that the value of PDMS is bigger than others. So, PDMS has the potential to improve the temperature sensitivity of fiber sensors.

### 2.1. A Variety of Structures Based on Mach–Zehnder Interference

In the traditional optical fiber sensing principle based on Mach-Zehnder interference, the light in the sensor can usually be divided into core-mode light (reference-mode light) and cladding-mode light (sensing mode light) [19,20]. The energy distribution of the light field can be analyzed according to the light intensity interference formula [20,21,22,23]:

(1) $$I=I_{1}+I_{2}+2\sqrt{I_{1}I_{2}}\cos\left(\Delta \varphi \right)$$

In the above formula, *I*_1_ and *I*_2_ are the descriptions of the core light intensity and cladding light intensity, respectively. Δφ is the optical path difference formed when light travels through different paths, at the same time; it can be expressed in another concrete form:

(2) $$\Delta \varphi =\frac{2\pi L\Delta n_{eff}}{\lambda }$$

It can be seen from the above equation that the effective length *L* and the effective refractive index difference $\Delta n_{eff}$ of the sensor have an important influence on Δφ. λ is the free space wavelength. In practice, the change of these two parameters will indirectly affect the sensing performance of the fiber. In the sensor test of parameters, such as temperature and the refractive index, *L* and $\Delta n_{eff}$ will be changed accordingly with the change of the parameters to be measured. For the effective length, when the fiber is combined with material, it is limited by both the fiber and utilized material. A comprehensive thermal expansion coefficient $\alpha_{eff}$ is used to describe the $\alpha_{fiber}$ of the fiber itself and the $\alpha_{material}$ endowed by the utilized material. When the measured temperature changes ΔT, the effective length changes as follows:

(3) $$\Delta L=\alpha_{eff}\Delta TL$$

The description of the effective refractive index is divided into two common cases: One is that the material is coated outside the cladding of the fiber, and the other is that the material is filled inside the fiber core. In the first case, the effective refractive index of cladding will be changed, while in the second case, the effective refractive index of the core will be changed. For two different cases, $\varepsilon_{1}^{}$, $\varepsilon_{2}^{}$, and $\varepsilon_{material}^{}$ are defined as the thermo-optic coefficient of the core, cladding, and material, respectively. When the material is filled into the core, $\varepsilon_{1}^{eff}$ is used to describe the effective parameters of $\varepsilon_{1}^{}$ and $\varepsilon_{material}^{}$. In the same way, $\varepsilon_{2}^{eff}$ is used to describe the effective parameters of $\varepsilon_{2}^{}$ and $\varepsilon_{material}^{}$ for the material coated outside the cladding [20]. Depending on the situation, the difference of the effective refractive index can be defined as:

(4) $$\Delta n_{eff}=n_{core}^{eff}-n_{clad}^{eff}=\left(\varepsilon_{1}^{eff}n_{core}-\varepsilon_{2}^{eff}n_{clad}\right)\Delta T$$

When the excitation of the optical fiber sensors is a broadband light source, the dips of the interference spectrum will appear at the particular location, and m is a positive integer:

(5) $$\lambda dip=\frac{2\Delta L\cdot \Delta n_{eff}}{2m+1}$$

The spectral drift is defined under the influence of many parameters:

(6) $$\Delta \lambda =\left[\left(\alpha_{eff}+\varepsilon_{1}^{eff}+\varepsilon_{2}^{eff}\right)\Delta T\right]\lambda$$

By coating the material on the optical fiber, the variables in the formula are modified, so as to improve the sensitivity of optical fiber sensing. This is the principle used in this part of the sensor. The performance of PDMS, which has a higher negative thermal optical coefficient (−4.66 × 10^−4^/°C) and thermal expansion coefficient (300 × 10^−6^/°C), has been fully reflected in the sensor [19].

**Figure 1:**
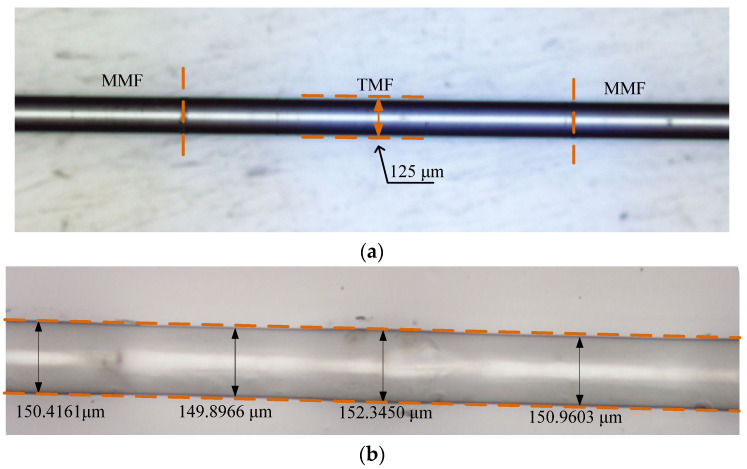
Microscopic images of sensors: (**a**) original fiber sensor. (**b**) PDMS-coated fiber sensor [20].

When PDMS is coated on the fiber cladding, in order to fully reflect the negative thermal optical coefficient characteristic of the PDMS, the underlying fiber structure needs to be able to excite strong evanescent waves. The core off-set fiber, tapered fiber, and bend fiber can excite a strong evanescent wave, and it is characterized by simple preparation, strong plasticity, and low cost, which are widely concerned [20,21,22,23,24]. A fiber structure composed of multi-mode fiber (MMF), thin-mode fiber (TMF), and multi-mode fiber was used to verify the improvement of temperature sensitivity of the fiber sensor by PDMS in [20]. Through several temperature experiments on the sensor before and after PDMS coating, it was found that the sensitivity of the temperature changed from 47.14 pm/°C to 75.04 pm/°C, and the sensitivity increased 1.6 times. Better temperature stability was shown through the long-term temperature test. In the literature, the fiber was suspended on a scaffolding and kept perpendicular to the ground. PDMS was added at the upper end of the sensing part with a glue head dropper. Under the action of gravity, PDMS flowed down the fiber, and PDMS was coated on the fiber. This coating process is very simple. The comparison of the original fiber, which is with just the cladding, and coated fiber is shown in Figure 1, where it can be found that the thickness of the PDMS film is relatively uniform, which fully demonstrates the simplicity of the PDMS coating process.

**Figure 2:**
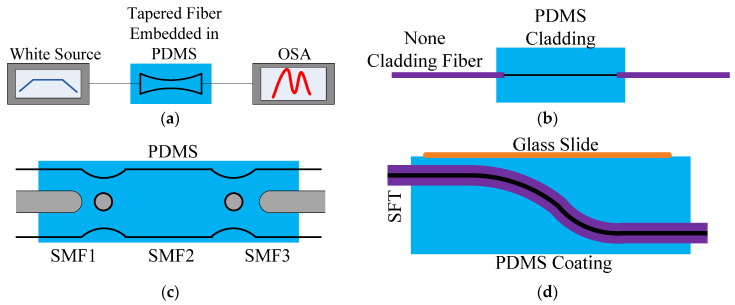
The fiber sensors coated with PDMS: (**a**) Tapered fiber embedded in PDMS [21]; (**b**) Core-fiber with PDMS cladding [22]; (**c**) Bowknot taper fiber coated with PDMS [23]; (**d**) S-shaped taper fiber coated with PDMS [24].

Various kinds of tapered fibers were made to measure different parameters. In [21], the tapered fiber was obtained by tapering the standard single-mode fiber (SMF) [21], which can be used to measure the temperature; the fiber is shown as Figure 2a. The cladding of a section of fiber is removed and the new cladding of the fiber is replaced by a thicker PDMS layer [22], as shown in Figure 2b. In [23], the bowknot-type taper was prepared by melting two single-mode fibers into balls. It is a deformation of the tapered structure, as shown in Figure 2c. In Figure 2d, a section of fiber was pulled into an s-shaped fiber taper (SFT) [24], giving it the ability to measure pressure by adding PDMS blocks throughout the tapered area.

The sensors of [21,23,24] were used to measure the temperature, and they can measure the pressure in [22,24]. Although the fiber structure and the measurement are different, PDMS plays a role in protecting the fiber, especially in the pressure test. A soft PDMS membrane is necessary for the fragile tapered structure.

**Figure 3:**
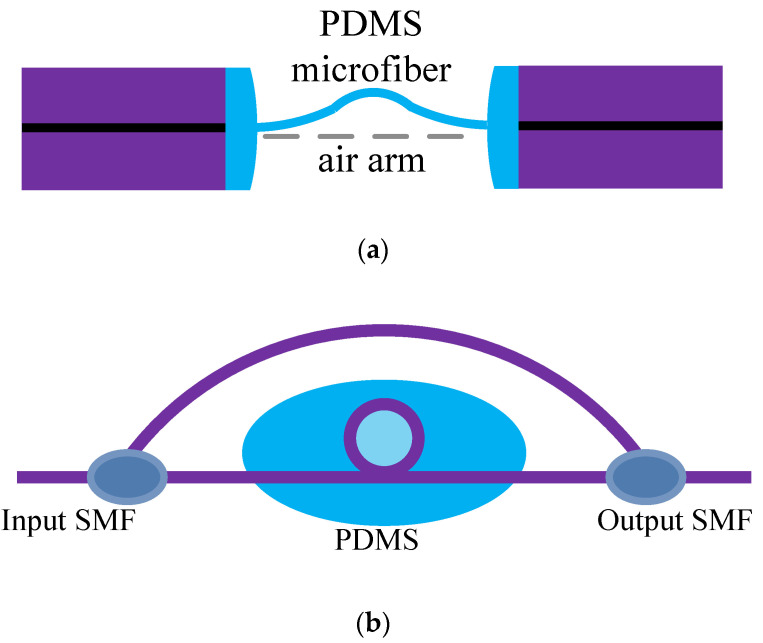
Applications of PDMS and Mach–Zehnder structure: (**a**) Sensor consists of the PDMS microfiber and air arm [25]; (**b**) Sensor consists of a bend arm and straight arm [26].

It is different from the all-fiber sensor based on the Mach–Zehnder interference. The Mach–Zehnder sensors in [25,26] completed the sensor arm and the reference arm. From Figure 3a, the PDMS microfiber was the reference arm, which transmitted light steadily, and the light intensity was relatively stable. Because the micro-nano fiber made by PDMS had good flexibility, it can be stretched in a certain range. With the increase of the distance between the two fiber ends, the light intensity in the air arm decreases, resulting in the change of the interference spectrum, so as to measure the micro-displacement [25]. A sensor with a real sensor arm is shown in Figure 3b. By coating the sensor arm with PDMS, it is made more sensitive to temperature sensing [26]. Different from the structure in [26], the temperature sensing experiment was completed in [27] with only one micro-nano fiber ring, and the sensitivity increased 9 times, from 183 to 1.67 nm/°C.

**Figure 4:**
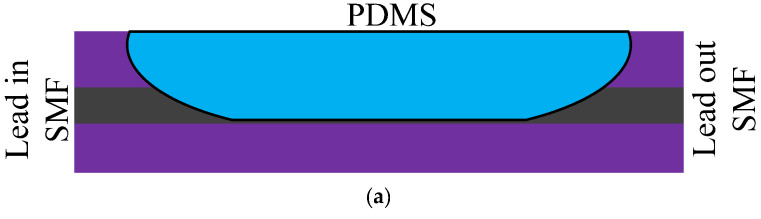

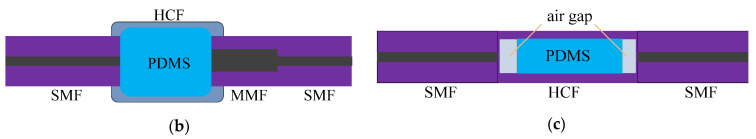
The sensors were filled with PDMS: (**a**) D-shaped fiber coated with PDMS [28]; (**b**) Hollow core fiber filled with PDMS [29]; (**c**) Hollow core fiber filled with air gap-PDMS-air gap [30].

In the literature described above, PDMS was coated outside the cladding of the fiber as a coating material. In [28,29,30], PDMS was filled into the fiber as a special fiber core. As you can see in Figure 4, a section of the cladding and core of the standard single-mode fiber was removed, and the damaged area of the fiber was filled with PDMS with a certain thickness [28]. PDMS acts as the cladding and core at the same time. In order to give the fiber structure greater mechanical strength, a segment of hollow core fiber (HCF) filled with PDMS can be connected within the sensor [29]. In a similar fiber optic structure, two air chambers are added to both ends of the PDMS [30]. At the same temperature change, the PDMS size has the potential to change even more, making it possible for the fiber to achieve higher sensitivity. However, this delicate manipulation is more difficult.

### 2.2. A Variety of Structures Based on FBG/LPFG (Long-Period Fiber Grating)

Among the many kinds of optical fiber sensor components, FBG has become the highest commercial optical fiber sensor due to its better stability and lower price. Although the stability of FBG in the sensor is far less than that of other sensors, the fact that its sensors are less sensitive cannot be ignored. Usually, methods of making FBG with special materials and coating the material outside FBG are selected to improve the sensitivity of the sensor [31,32,33,34]. The sensor principle of FBG is also discussed from two aspects, stress and temperature. 

The Bragg resonance wavelength ($\lambda_{B}$) will appear in the following position [32]:

(7) $$\lambda_{B}=2n_{eff}\Lambda$$

In the above equation, $n_{eff}$ and Λ represent the effective refractive index of the core and grating pitch, respectively. Obviously, $n_{eff}$ and Λ are the two main factors affecting the sensitivity. They will change according to the external environment; the related formula can be obtained [32,33,34]:

(8) $$\frac{\Delta \lambda_{B}}{\lambda_{B}}=\frac{\Delta n_{eff}}{n_{eff}}+\frac{\Delta \Lambda }{\Lambda }$$

When FBG is subjected to external strain, the grating constant changes as the length of FBG becomes longer, and the refractive index of FBG changes under the elasto-optical effect. A classic description is summed up to describe this change, where $P_{e}$ is represented by the photoelastic constant, and ε is represented by the strain induced:

(9) $$\frac{\Delta \lambda_{B}}{\lambda_{B}}=(1-P_{e})\varepsilon$$

In the temperature test, the grating constant and refractive index of the grating are affected by the thermal expansion coefficient (α) and thermo-optic coefficient (ξ), respectively. The total effect of the temperature on the wavelength drift is as follows:

(10) $$\frac{\Delta \lambda_{B}}{\lambda_{B}}=(\alpha +\xi )\Delta T$$

After a comprehensive treatment of temperature and strain, a comprehensive formula is summed up:

(11) $$\frac{\Delta \lambda_{B}}{\lambda_{B}}=(1-P)\varepsilon +(\alpha +\xi )\Delta T$$

Whether PDMS is coated outside FBG or filled inside FBG, it can be discussed through the four aspects in the above equation.

**Figure 5:**
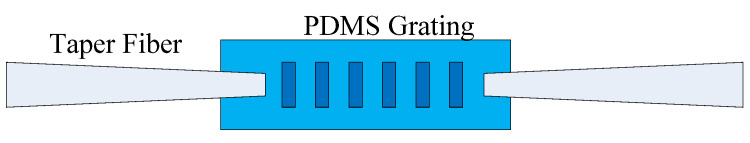
The schematic diagram of LPFG made up of PDMS [31].

A kind of LPFG composed of tapered fiber and PDMS grating was made [31], as shown in Figure 5. Before processing, the tapered fiber was passed through the mold with a periodically recessed structure. The PDMS was injected into the mold, in order to ensure that PDMS had good fluidity and could fill the entire mold, and the temperature was kept at 110 °C for 10 min. After the PDMS solidified, the outer mold was removed, and one LPFG was finished. For the current high-precision processing technology, this process is very simple, and the PDMS grating produced by this method has good controllability in the micron size. In the temperature test, a temperature sensitivity of −1.328 nm/°C was shown, which is not possible for conventional LPFG.

**Figure 6:**
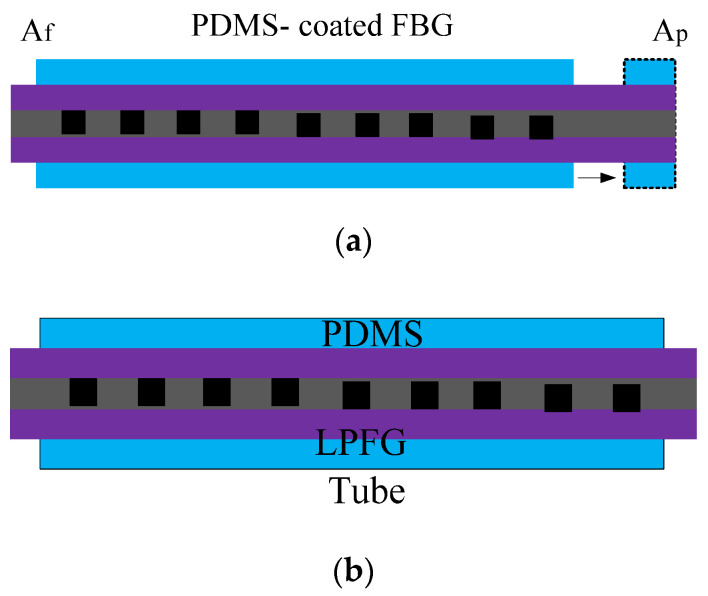
The schematic diagram of sensors: (**a**) FBG sensor coated with PDMS [32]; (**b**) LPFG with a tube filled with PDMS [33].

Different from the preparation method in [31], the method of coating PDMS on the outer layer of the sensor is more acceptable. In [32,33], PDMS was selected to increase the temperature sensitivity. As expected, the temperature sensitivities of the FBG and LPFG could be effectively improved by 4.2 times [32] and 4 times [21], respectively. Comparing the principles of Figure 6a,b, the functionality of PDMS is different. The light field is distributed only in the fiber core for FBG. Therefore, when the temperature increases, the size of PDMS will become longer and the length of FBG will increase. *A*_f_ and *A*_p_ represent the starting position and end position of the PDMS jacket after expansion [32], respectively. For the LPFG, there is part of the light intensity in the cladding. Under the effect of the evanescent wave, light waves will interact with the external materials. Therefore, when considering the effect of thermal expansion on the size of LPFG, the effect of PDMS on the effective refractive index of cladding should also be considered [33]. In [34], an FBG coated with PDMS was designed and manufactured to detect chemical reagents. Because of the selective tensile effects of the PDMS, in different volatile organic compound (VOC) solutions, the length of PDMS will change, which will provide power for the size change of FBG [34]. This provides a new idea for the application of PDMS.

### 2.3. A Variety of Structures Based on F-P

According to the characteristics of PDMS, it is usually used to change the effective sensing length and effective refractive index of P-F sensors. PDMS is generally coated at the end of sensors or filled into the cavity, because it adopts the sensing mode of endpoint measurement [35,36,37,38,39].

**Figure 7:**
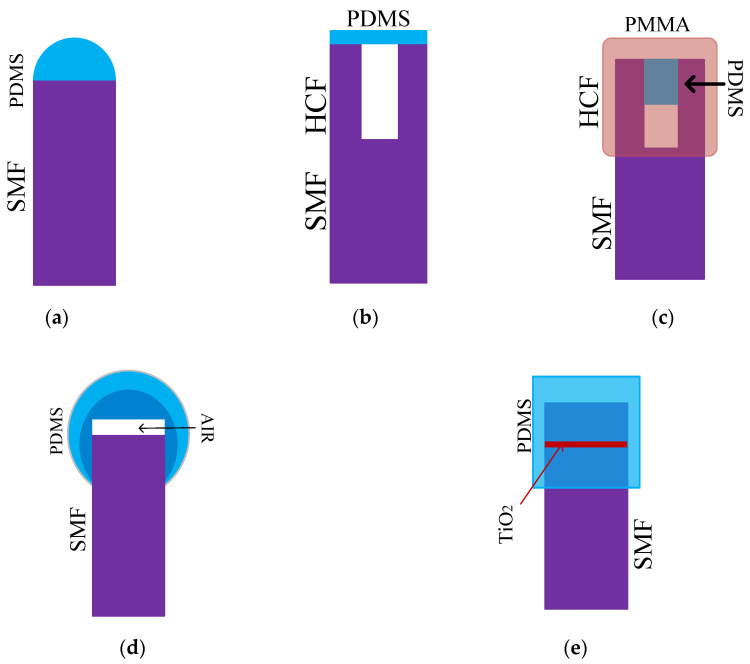
The descriptions of different F-P sensors: (**a**) The F-P sensor consists of SMF and PDMS [35]; (**b**) F-P sensor consists of SMF, HCF, and PDMS [36]; (**c**) F-P consists of SMF, HCF filled with PDMS and a PMMA (polymethylmethacrylate) protective layer [37]; (**d**) Sensor structure consists of SMF, air cavity, and two-PDMS layers [38]; (**e**) Sensor consists of SMF with the TiO_2_ layer and PDMS [39].

Five F-P sensors with a similar structure are summarized for comparison in Figure 7, and their infrastructure is a standard single mode fiber (SMF). In [35], PDMS was dropped at the end of SMF and formed a hemispherical structure when PDMS solidified; the sensor is shown as Figure 7a. After leaving the SMF, the light in the fiber core will continue to spread in PDMS and will be emitted on the basic surface of PDMS and air, and the forward-propagating light will interfere with the back-propagating light. When the temperature changes, the length and refractive index of PDMS change, and the position of the interference wavelength changes significantly, while the peak intensity does not [35]. A segment of the hollow core fiber (HCF) was fused to SMF and a layer of PDMS film was applied to the end of the HCF [36], which is shown as Figure 7b. With the same function as PDMS in [34], the sensors were used to measure VOCs.

Different from the structure of [36], PDMS was filled into the core of a hollow silica capillary (HSC) to prevent PDMS leakage, and polymethylmethacrylate (PMMA) was applied to the outside of the sensor for sealing [37]. In the temperature range of 35 to 45 °C, its sensitivity can be up to 1.509 nm/°C. In Figure 7c, the positions of PDMS and PMMA are clearly marked.

An air cavity was also applied to the F-P structure in [38]. Instead of using HCF directly, a hollow will be created by heating the paraffin, which is coated between the optical fiber and PDMS [38]. In Figure 7d, two layers of PDMS can be seen, where the outer PDMS acts to protect the sensor. The length of PDMS will change after adsorption of toluene vapor. In [39], a thin layer of TiO_2_ was coated on one port of fiber, before two SMFs were fused, and an F-P structure was introduced into the fiber. TiO_2_ (red section) is shown in Figure 7e. Through the analysis of the above examples, it can be found that PDMS not only plays a role in temperature sensing but is also widely used in chemical measurement.

### 2.4. A Variety of Structures Based on SPR (Surface Plasmon Resonance)

**Figure 8:**
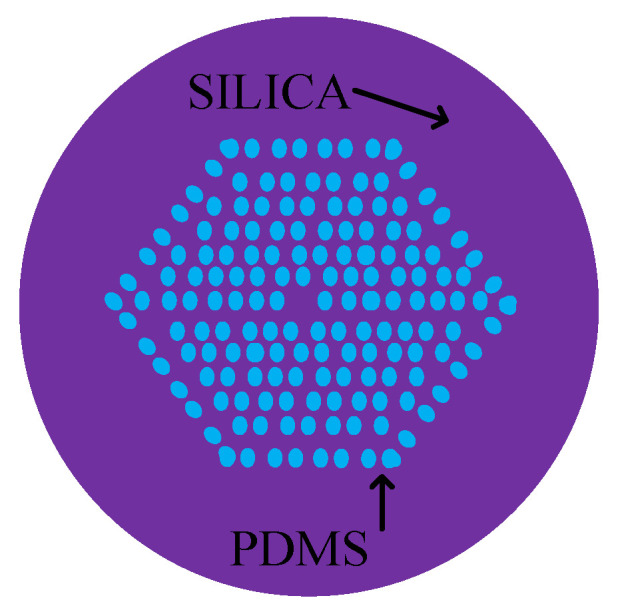
The schematic diagram of PCF filled with PDMS [41].

In the above fiber-optic sensor example, we have seen that PDMS is widely used but PDMS is not a panacea. In order to meet the needs of optical fiber sensing, we can choose other materials for replacement, and we can also combine PDMS with other materials to improve the sensing performance of PDMS [40]. Especially for fiber optic sensors based on SPR, the combination of metal materials and PDMS is very important [41,42,43,44]. PCF is used in sensors due to its inherent air hole and good sensing performance. PDMS was filled into the air holes to improve the ability to reduce the energy loss with the temperature rising [41]. The micrograph is shown in Figure 8.

**Figure 9:**
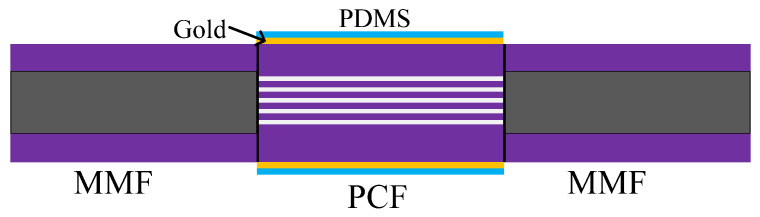
The description of PCF coated with PDMS based on SPR [42].

In [42], in order to excite the evanescent wave required for the SPR principle, the PCF was fused in the middle of the MMF. At the same time, gold and PDMS were coated on the PCF in turn [42]. The evanescent wave generated by the SPR effect can penetrate the metal layer and interact with PDMS, where the change of the refractive index of PDMS played a major role in the sensing as the fiber length was limited by gold, which is shown as Figure 9. The temperature sensitivity was −1.551 nm/°C.

**Figure 10:**
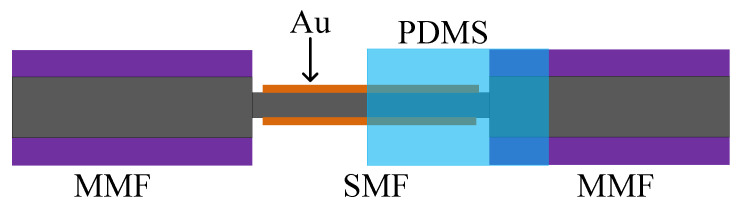
The description of the SPR sensor coated with Au and PDMS [43].

A sensor with a relatively simple MMF-SMF-MMF structure was applied to the SPR sensors [43,44]. Similar to the sensor in [42], Au was coated outside the SMF to excite SPR. What is interesting is that PDMS was only applied to half of the SMF; the description is shown as Figure 10. The sensor coated with PDMS was sensitive to temperature while, at the same time, the bare sensor was better at detecting the refractive index. The temperature and refractive index can be measured simultaneously [43]. In [44], graphene-gold-Au@Ag NPs-PDMS were coated on a sensor of the same MMF-SMF-MMF structure. In this work, the graphene-gold-Au@Ag NPs showed an ability to excite SPR as a special metal, and PDMS was used to increase the temperature sensitivity [44]. More importantly, a good ability to combine with other materials was fully demonstrated.

### 2.5. SPR Sensors Based on LPFG

A necessary mechanism for the SPR effect is to have a sufficiently strong evanescent wave to excite the SPW (surface plasmon wave). When the propagation constant of the light wave in the cladding matches that of the SPW, the energy of the cladding mode will be converted into the energy of SPW, so that the light energy of the corresponding band of the transmission spectrum will suddenly drop and a resonance absorption peak will appear, whose position is the SPR resonance wavelength [45,46,47]. Therefore, the fiber optic sensor needs to be configured with a special structure to stimulate a strong evanescent wave, which is described in Section 2.4. However, the special fiber structure will not only destroy the mechanical strength of the fiber but also increase the difficulty of sensor preparation. LPFG with an original characteristic of the strong evanescent wave is more suitable to be combined with the SPR principle [45,46,47,48].

According to the basic principle of LPFG, the position of the light intensity and the interference spectrum of the evanescent wave can be controlled by adjusting parameters, such as the grating period. For the LPFG sensors, there are many active modes of light within the cladding that interact with the material coated outside the cladding. Different from the plate-SPR principle, SPR is considered as a new mode outside the cladding for LPFG-SPR.

**Figure 11:**
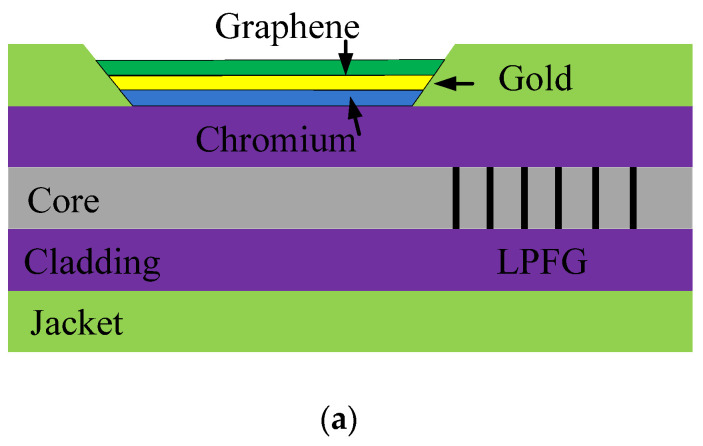

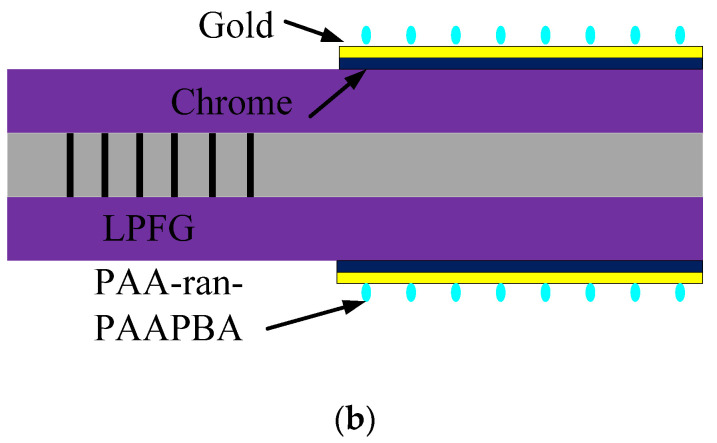
The description of the SPR sensors: (**a**) The jacket of LPFG is side-polished and coated with multilayer materials [49]; (**b**) LPFG sensor coated with chrome, gold, and the functional material [50].

An SPR sensor based on side-polished LPFG was applied to continuous glucose monitoring in [49]; the concrete schematic diagram can be found in Figure 11a. At the same time, the similar fiber structure is shown in Figure 11b in [50]. In [49], the composite metal thin layer of chromium, gold, and graphene was used to excite the SPR; however, the metal layer was replaced by chrome and gold in [50]. The biocompatible borate polymer, PAA-ran-PAAPBA, was coated on the metal layer part of the two sensors. The purpose of using multiple layers of metal is to get a stronger SPW and the PAA-ran-PAAPBA is a carrier that can be specifically identified with glucose, while the sensors have the function to monitoring the glucose [49,50].

**Figure 12:**
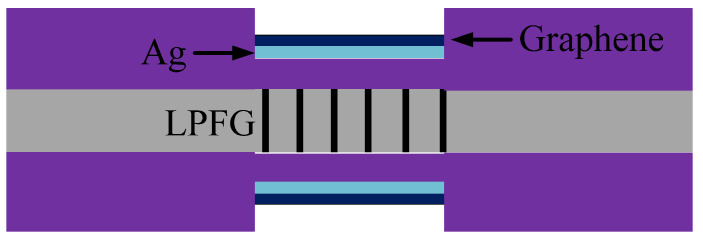
The gas sensor based on SPR-LPFG with Ag and graphene [51].

In [51], in the gas sensor shown in Figure 12, part of the cladding on LPFG was removed. This led to a more intense evanescent wave because the light path was reduced, and Ag and graphene were used to excite the SPR. Because the surface of graphene is loose and porous, the gas can act well on the SPP. So, the sensor has a high sensitivity of the gas. In this study, the sensitivities of LPFG, LPFG + Ag, and LPFG + Ag + graphene were compared, and the result showed that the sensitivity of LPFG + Ag + graphene was much higher than the other two cases [51].

**Figure 13:**
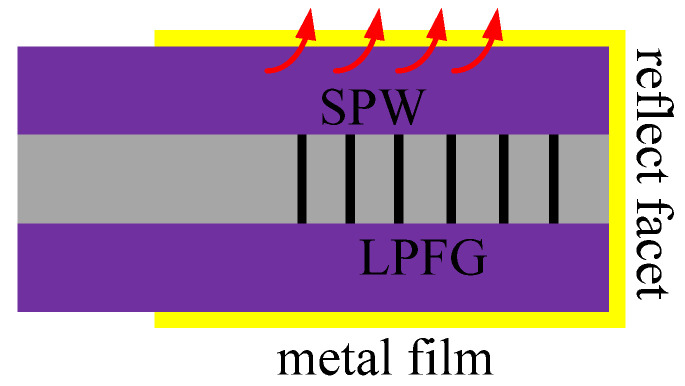
The structure of the reflected LPFG-SPR sensors [52,53].

Novel reflected SPR sensors based on LPFG were proposed in [52,53], the schematic diagram is shown in Figure 13. They were different from the two SPR sensors mentioned above, as this type of sensor not only needs to coat the cladding with metal material but also needs to ensure that the fiber cross-section is flat enough to ensure that enough light is returned to the OSA. This sensor can be used in a wide range of exploration fields due to its more flexible and compact structure [52,53]. In the experiment, there is a noticeable spectral drift of different concentrations of alcohol.

## 3. Sensors Coated with Polyimide, Acrylate, and Materials for Cryogenic Applications

Unlike PDMS, which is mostly used for temperature measurement, polyimide is mostly used for humidity measurement while measuring temperature. With a strong structural strength, polyimide can still exist stably in an environment of 400 °C and can be used for a long time in the temperature range of −200 to 300 °C [54,55,56,57,58,59,60,61,62,63,64,65,66,67,68,69,70,71,72,73,74,75,76].

The application of sensors coated with different materials is becoming more and more abundant at cryogenic temperatures. It is worth discussing the coating materials to improve cryogenic temperature sensitivity. In [54], fiber sensors coated with different coating materials, such as acrylate, tin, indium-bismuth, and lead-tin, were described in the temperature range from 4.2 to 61 K. The results showed that the fiber coated with indium-bismuth had minimal sensitivity variation compared to the others [54]. Aluminum and PMMA, which are two contrast materials, were applied on FBG to verify the temperature sensitivities in the temperature range from 80 to 300 K [56]. The temperature sensitivity of the PMMA substrate, which was 0.04 nm/K, was twice as large as that of the aluminum substrate at 100 K. In [57], the sensitivities of bare FBG, acrylate-coated FBG, and polymer-coated FBG were compared and analyzed at temperatures from −180 to 25 °C [57]. The FBG coated with polymer had the biggest value of 48 pm/°C, which was 10 times bigger than that of the bare FBG. The same type of experiment was conducted in [60], where different materials, such as Ni, Cu, Zn, and Sn, were coated on the FBGs [60]. The temperature sensitivities of them were 1.5 times, 2 times, 2.5 times, and 3 times more than that of the bare FBG, respectively [60]. Equally important, the structures of fiber sensors were not damaged under the cryogenic temperatures [54,62,63]. It is an essential advantage for fiber sensors to be applied in the field of cryogenic measurement.

When the fiber is bent, not only the loss of light intensity will increase but also the structure of the fiber will become weak. At very low temperatures (77 K), the fiber made up of SiO_2_ will become very fragile. This is disastrous for fiber optics applications in ultra-low temperatures [64]. In [64], polyimide was coated on the outer layer of the curved fiber to improve the frost resistance of the fiber. The stability of the optical fiber sensor in a high-temperature test was discussed by the sensor coated with polyimide, copper, aluminum, or gold [75]. By analyzing the experimental results in detail, we found that polyimide and copper had good stability. However, copper oxidizes easily at high temperatures, thus polyimide is the best option. An FBG was made by the fiber, which was made up of polyimide, to measure the temperature in the range of 30–180 °C [76].

**Figure 14:**
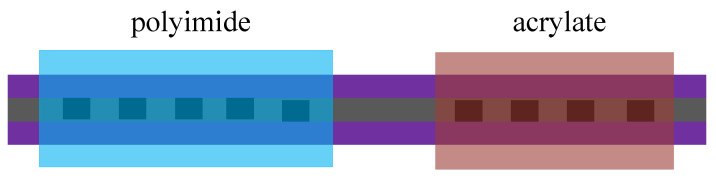
The FBG sensor coated with polyimide and acrylate [77].

In practical applications, there are many parameters to be measured, and the method of cascading two FBGs is used to obtain a stronger sensing capability [77]. As shown in Figure 14, a sensor consisting of two FBGs was used to measure temperature and salinity. When salinity changes, the water absorption capacity of polyimide will change, leading to a change in the length of the sensor. So, the FBG coated with polyimide was sensitive to both temperature and salinity. However, the acrylate cannot absorb water and was only sensitive to the temperature. The sensor can compensate for salinity while measuring the temperature. In [78], the polyimide was also coated on FBG for the temperature test. For FBG with the same parameters, different thicknesses of polyimide will also change the measurement results [79]. The effect of the polyimide film thickness on temperature sensing was discussed in [79]. Coated FBGs with effective diameters of 170 and 143 um were tested in a radiation hard humidity range of 0 to 100. To ensure the accuracy of the results, the experiments were completed at 20, 0, and −15 °C, respectively. It was found that the thicker sensor had a higher sensitivity.

**Figure 15:**
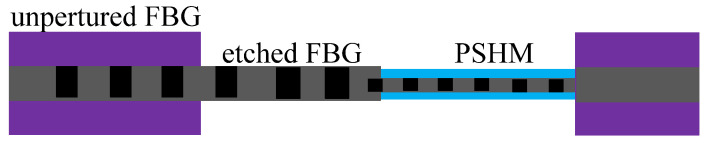
The configuration of the FBG with PSHM [80].

A composite structure of FBG was produced to complete the measurement of cell growth and temperature in [80]. A complete FBG was partially etched and a polyimide silica hybrid membrane (PSHM) was coated on the etched FBG [80]. The configuration of the FBG is shown in Figure 15. Compared with the sensor of [77], the structure of [80] was compacted; however, the process was more difficult.

**Figure 16:**
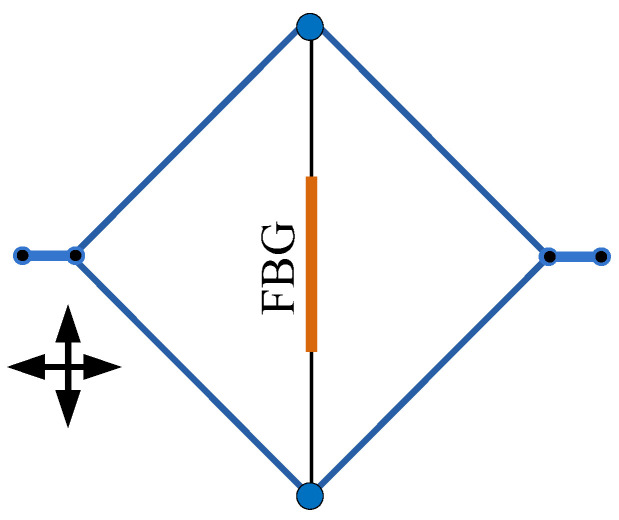
The schematic diagram of polyimide-FBG installed in the rhombus metal structure [81].

In [81], an FBG coated with polyimide was installed in the rhombus metal structure; the relative positions of FBG and rhombus are shown in Figure 16. The sensor was used for the strain at a high temperature, and the function of polyimide was the stability sensing performance [81], which is the same as that in [75].

When sensing, the change of the refractive index and the length of polyimide are the main factors used to enhance the sensing performance. Adding different compounds to polyimide will greatly improve its basic performance. Various pore-foaming agents, such as lithium chloride, acetone, methyl alcohol, and activated carbon, have been added into the polyimide to explore the effects on sensitivity in [82,83]. By comparing the sensitivities of different doped materials, the sensitivity of a sensor coated with polyimide-lithium chloride was nearly three times that of the other sensors [82].

**Figure 17:**
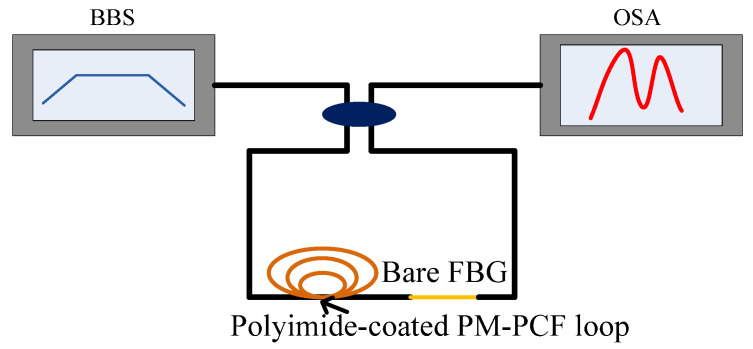
The sensor consists of FBG and salinity fiber [84].

**Figure 18:**
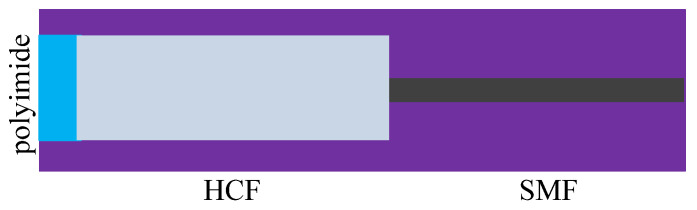
The configuration of the F-P consists of SMF, HCF, and polyimide [85].

In [84], a salinity sensor consisting of a PCF loop coated with polyimide was used to measure the temperature and liquid, which is shown in Figure 17. The experimental results show that the sensitivity of salinity, which was 0.742 nm/(mol/L), was 45 times than that of FBG coated with polyimide [84]. When polyimide absorbs water, the volume increase will generate pressure in the radial direction of the loop. Because the length of the loop was 20.8 cm, which was larger than the traditional sensor, the sensitivity of salinity was ideal. The FBG was only sensitive to temperature, and it can compensate for the temperature. An F-P sensor consisting of SMF, HCF, and polyimide membrane is shown in Figure 18. When the salinity is changed, the length of the air chamber will change by the shrinkage of polyimide [85]. The influence of temperature on the interference spectrum can be removed by adjusting the length of HCF, so a temperature-independent fiber salinity sensor is thus successfully produced.

**Figure 19:**
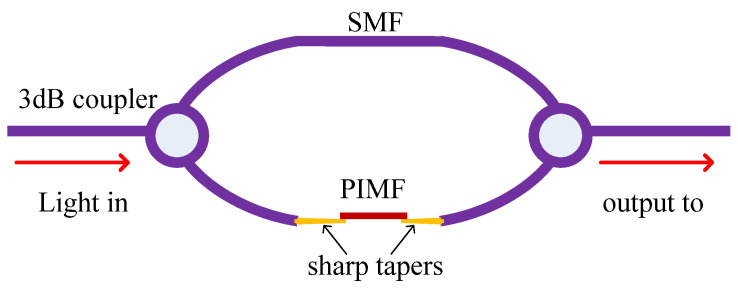
The MZI sensor based on PIMF and SMF [87].

An MZI sensor was proposed by welding SMF, MMF, and SMF in turn [86], and the sensor had a stable sensitivity of 0.0115 nm/°C in the range of 30–300 °C by coating polyimide on the sensor. In Figure 19, two 3-dB couplers are connected together by SMF and PIMF (polyimide microfiber) [87]. PIMF was connected at both ends of sharp tapers as an amplifier for temperature and salinity. When the length of PIMF was changed, the spectrum of interference changed accordingly. At the same time, the sensitivities of different diameters/length of PIMF were discussed, and sensitivity increased as the diameter/length increased. This suggested that a larger PIMF is more responsive to measurement.

## 4. Sensors Coated with UV-Sensitive Materials

**Figure 20:**
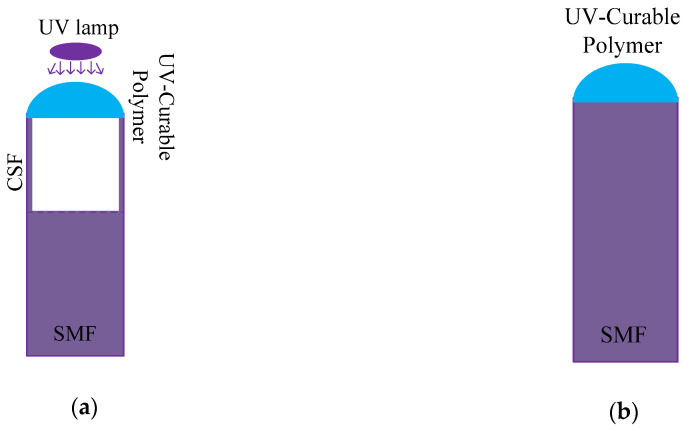
The description of the structures: (**a**) The lensed fiber consists of SMF, CSF, and UV-curable polymer [90]; (**b**) Sensor consists of SMF and UV-curable polymer [91].

Compared with other materials, the fabrication of coating UV-curable polymer on the fiber is relatively simple, because UV-curable polymer has the characteristic of curing when exposed to UV light [88,89,90,91,92,93,94]. In the absence of UV light at room temperature, UV-curable polymer can maintain the state of the liquid for a long time, which makes it possible to manipulate it on sensors. When UV-curable polymer is irradiated by UV light, the volume of it will shrink with the curing process. So, the shrinkage of cured volume was explored in [88]. A lensed fiber was proposed, which consisted of SMF, CSF (coreless silica fiber), and a hemisphere of UV-curable polymer. The description is shown in Figure 20a.

The fabrication is similar to that in [35], where the F-P sensor based on SMF was used again. The sensors of Figure 20 were tested in [90,91], where the difference is that the sensor was used to measure the temperature and refractive index in [81], while it was used to measure temperature and pressure in [91]. This fully shows the potential of UV-curable polymer in fiber sensing.

**Figure 21:**
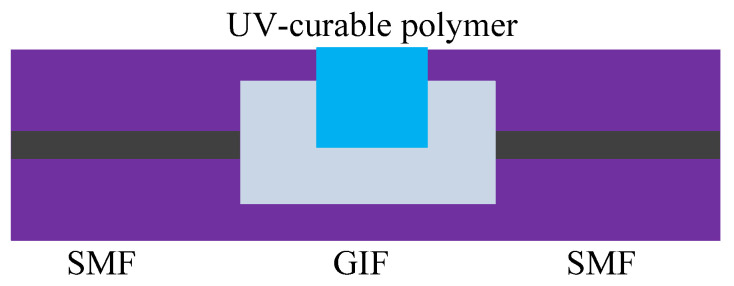
The MZI sensor based on GIF and UV-polymer [92].

A compact M-Z sensor was developed by fusing SMF and graded index fiber (GIF) [92]. From Figure 21, it can be found that GIF has a tiny cavity filled with UV-curable polymer. When light propagates in GIF, due to the different refractive indexes of GIF and UV-curable polymer, the optical path of the two parts is different, and an interference phenomenon will occur. Through many experiments, it was found that the sensor has a good stability, which is also the embodiment of UV-curable polymer’s performance. In order to fully understand the performance of UV-curable polymer, various elements were doped in it for temperature experiments. A PCF filled with the mixture of CdSe/ZnS quantum and UV curing adhesive was demonstrated in [93].

**Figure 22:**
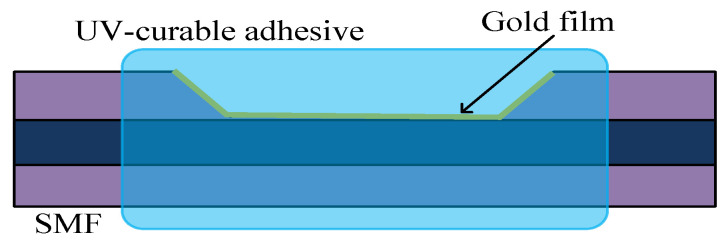
The SPR sensor consists of UV-curable adhesive and gold film [94].

The combination of UV-curable adhesive and SPR is indispensable in many kinds of sensors. In [94] and Figure 22, an SPR sensor was proposed based on SMF, UV-curable adhesive, and gold film. The whole sensing area was completely enclosed by UV-curable adhesive, which enhanced the sensing function while protecting the fiber structure. To ensure the accuracy of the temperature sensitivity, experiments were carried out at 25 to 100 °C and 100 to 25 °C.

## 5. Sensors Coated with Graphene, Metal Ions, and Others

In recent years, different kinds of graphene and metal ions have been widely studied and used in optical fiber sensors. Many sensors, although coated with different materials, are based mostly on SPR principles [95,96,97,98,99,100,101,102,103,104,105,106,107,108,109,110,111,112,113,114,115,116,117,118,119]. Because the effective surface area of the optical fiber is small, it is difficult to coat the material on the optical fiber. A prism coated with material was used in the SPR sensor [95,96,97]; at the same time, it is the original structure based on SPR. However, in prism experiments, the instruments are more expensive and the process more complex. Once again, the focus of the experiment goes back to the fiber.

**Figure 23:**
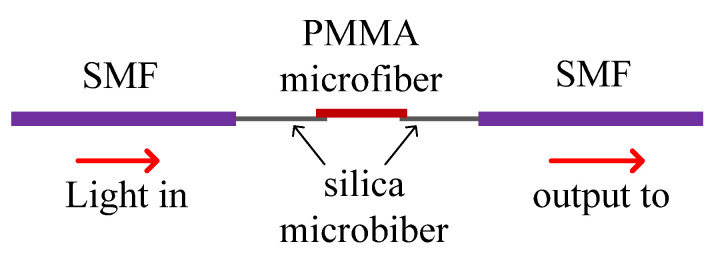
The sensor based on the PMMA microfiber [104].

For sensors coated with graphene, in [99], a U-shaped sensor coated with graphene/AgNPs was proposed by SPR [99]. In [100], the cladding of SMF was removed, the graphene oxide encapsulated gold nanoparticle was coated on the core, and a novel cladding was made [100]. A structure that was similar to that in [28,94] was mentioned again, except that the coated material became graphene. Without exception, such sensors are also structurally weak. To solve this problem, a section of HCF internally filled graphene quantum dots were used to strengthen the optical fiber structure [102]. The approach is also suitable for sensors that use alcohol as a sensor. The PCF filled with alcohol was proposed to avoid alcohol evaporation [103]. In [104], a microfiber made up of poly methyl methacrylate (PMMA) was connected with silica microfiber as shown in Figure 23. Under the evanescent wave, the light in the silica microfiber will be transmitted to the PMMA microfiber. When the temperature changes, the interference spectrum will vary with the length of the PMMA microfiber.

With the development of coating technology and fiber processing methods, more and more materials, such as ITO [107], TiO_2_ [108], gold nanoparticles [109,110,111,112,113], and ZnO [118,119], have been applied to fibers. In addition to the usual temperature and refractive index measurements, different chemicals were added to the coating material to enable the sensor to gain specific recognition. For example, the silver nanoparticles/PVP/PVA hybrid was used to measure ammonia [115].

In Table 2, the sensors mentioned above are classified according to the different coating materials and fiber structure. The serial numbers of the corresponding documents are filled in the form. According to the number of studies, we can find that PDMS materials and sensors based on MZI and FBG/LPFG are widely used.

## 6. Conclusions

In this review, fiber sensors based on different structures and materials were summarized and analyzed. Through the analysis of the sensing principle, it can be found that most coated materials have a large thermal expansion coefficient and thermal light coefficient. When the amount of temperature change is the same, the coated material can provide the sensor with a greater change of effective parameters, thus enabling the sensor to obtain a higher sensitivity. For the fiber sensor that needs to be coated, the performances of the material are very important, and the stability and reliability of the fiber structure are equally important. So, sensors based on FBG/LPFG are more widely used, while FBG/LPFG have better production technology and theoretical analysis. The same fiber structure with different coating materials will be given different sensing properties, and the combination of fiber and material is therefore flexible. However, it is not necessary to make the fiber structure with a fragile structure in order to better excite the material performance. In the future, more varieties of fibers coated with materials will be produced. With the development of chemical materials, materials with specific recognition functions will be applied to fiber sensors, which will bring the application of optical fiber sensing to a new milestone.

## Figures and Tables

**Table 1:** The thermal expansion coefficients of materials at room temperature.

Materials	Thermal Expansion Coefficient (/°C)
PDMS	300 × 10^−6^
AL	23.03 × 10^−6^
Ti	8.35 × 10^−6^
Au	14 × 10^−^^6^
polyimide	20 × 10^−^^6^–30 × 10^−^^6^
SiO_2_	0.55 × 10^−6^

**Table 2:** A conclusion of the sensing function and sensitivities.

	MZI	SPR	F-P	FBG/	PCF
				LPFG	
PDMS	20	29	35		41
	75.04 pm/°C	42	−240.425 dB/RI	32	
	21	−1.551 nm/°C	385.46 pm/°C	0.042 nm/°C	
	3101.8 pm/°C	4613.73 nm/RIU	36 (VOCs)	33	
	22	(Refractive Index Unit)	1.17 pm/ppm	255.4 pm/°C	
	applied pressure	43	(Parts Per Million)	34	
	23	2323.4 nm/RIU	37		
	0.1957 nm/°C	−2.850 nm/°C	1.509 nm/°C		
	24	44	38 (toluene)		
	−29.03 nm/N 2.17 nm/°C	−1.02 nm/°C	1.4 nm/g.m^3^		
	25	1224 nm/RIU	39		
	applied		0.13 dB/°C		
	displacement				
	26				
	−41.58 pm/°C				
	27				
	1.67 nm/°C				
	28				
	−0.4409 nm/°C				
	29				
	580.6 pm/°C				
	30				
	−384 pm/°C				
polyimide	86		84	76	
	0.0115 nm/°C		85	12.7 pm/°C	
	87		0.45 nm/(mol/L)	1.2 pm/µε	
	0.09648 nm/°C		(salinity)	77	
	60.5 pm/‰			0.0094 nm/°C	
	(salinity)			0.0165 nm/M	
				(salinity)	
				78	
				79	
				80	
				1.97 mmol/L/h	
				(cell growth)	
				81	
				1.814 pm/με	
				82	
				1.71 pm/%RH	
				(humidity)	
				84	
				0.742 nm/(mol/L)	
				(salinity)	
UV-sensitive materials	92	94	90		93
	24611.54 nm/RIU	8800 nm/RIU	0.19 nm/°C		0.057 nm/°C
	−13.27 nm/°C	−0.978 nm/°C	260 dB/RIU		
			91		
			249 pm/°C		
			1130 pm/MPa		
graphene	96	49		49, (glucose)	
	8.25 × 10^2^/RIU	51		51, (gas sensors)	
	97	102		0.344 nm%^−1^	
	99	123.7 pm/°C			
	100				
	2.449 ∆A/RIU				
	(sucrose)				
	101				
alcohol					103,
					6.6 nm/°C
methyl methacrylate	104				
	58.5 pm/°C				
Sol-gel derived Ti, SiO_2_				105	
				1067.15 nm/RIU	
Au-Ta_2_O_5_-Pd/Au		50		50 (glucose)	
		106 (hydrogen sensor)		113	
		109		−17.93 nm/RIU 37.31 dB/RIU	
		110			
		111			
		765 nm/RIU			
		112			
TiO_2_	107	45	108,	45,	
	5.4 RH/nm	(DNA aptamer)	69.38 dB/RIU	(DNA aptamer)	
	(humidity)				
Ag/PVP/PVA		51		51	
		52		52	
		114			
		67.6 nm/RIU			
		115			
		0.9 counts/ppm			
		(ammonia)			
Zn/ZnO				117	
				49.59 pm/°C	
